# A program evaluation reporting student perceptions of early clinical exposure to primary care at a new medical college in Qatar

**DOI:** 10.1186/s12909-021-02597-9

**Published:** 2021-03-17

**Authors:** Tanya Kane, Tawanda Chivese, Ayad Al-Moslih, Noora A. M. Al-Mutawa, Suhad Daher-Nashif, Nehdia Hashemi, Alison Carr

**Affiliations:** 1 Department of Population Medicine, College of Medicine, QU Health, Qatar University, Doha, Qatar; 2 Department of Clinical Academic Sciences, College of Medicine, QU Health, Qatar University, P.O. Box 2713, Doha, Qatar; 3 Primary Health Care Corporation, Doha, Qatar

**Keywords:** Medical education, Early clinical exposure, Early clinical experience, Primary health centre placements, Qatar, Middle East, Evaluation, Student perceptions

## Abstract

**Background:**

Though common practice in Europe, few studies have described the efficacy of early clinical exposure (ECE) in the Middle East. The barriers to clinical learning experienced by these novice medical students have not been reported. This evaluation reports on introducing ECE in primary care, supported by Experiential Review (ER) debriefing sessions. The evaluation explores students’ experiences of their acquisition of clinical and non-technical skills, sociocultural issues commonly encountered but underreported and barriers to clinical learning experienced.

**Methods:**

We conducted a cross-sectional study of three student cohorts in 2017–19: All second and third-year students at the new College of Medicine were invited to participate. The primary outcome was students’ perceptions of the aims of the Primary Health Centre Placement (PHCP) programme and how it facilitated learning. Secondary outcome measures were students’ perceptions of their learning in ER sessions and perceived barriers to learning during PHCPs. Student perceptions of the PHCPs were measured using a Likert scale-based questionnaire.

**Results:**

One hundred and fifty-one students participated: 107 in year 2 and 44 in year 3; 72.3% were female. Overall, most students (> 70%) strongly agreed or agreed with the purposes of the PCHPs. Most students (71%) strongly agreed or agreed that the PCHPs allowed them to learn about patient care; 58% to observe doctors as role models and 55% to discuss managing common clinical problems with family physicians. Most students (year 2 = 62.5% and year 3 = 67%) strongly agreed/agreed that they were now confident taking histories and examining patients. Student barriers to clinical learning included: Unclear learning outcomes (48.3%); faculty too busy to teach (41.7%); lacking understanding of clinical medicine (29.1%); shyness (26.5%); and finding talking to patients difficult and embarrassing (25.8%). Over 70% reported that ER enabled them to discuss ethical and professional issues.

**Conclusions:**

Overall, our Middle Eastern students regard ECE as beneficial to their clinical learning. PHCPs and ER sessions together provide useful educational experiences for novice learners. We recommend further exploration of the barriers to learning to explore whether these novice students’ perceptions are manifesting underlying cultural sensitivities or acculturation to their new environment.

**Supplementary Information:**

The online version contains supplementary material available at 10.1186/s12909-021-02597-9.

## Background

The globalisation of medical education continues to accelerate, [1–3] rendering it important to evaluate the implications of culture on different curricular components [4]. Although novice medical students have been shown to encounter mixed experiences during their first exposures to the clinical environment, the importance of early clinical exposure (ECE) in medical student training is well documented [5]. In addition to the perceived benefits, previous studies from European and North American contexts report several challenges students face in dealing with the sense of uncertainty and role confusions [6]. Although early clinical placements in primary healthcare are common in European undergraduate medical programmes, [7] few studies have described the efficacy of ECE in the Middle East and the barriers to clinical learning experienced by young students. Established as the state’s first indigenous medical school, the College of Medicine (CMED) at Qatar University admitted its first cohort in 2015, the majority self-identifying as Arab. The 6-year undergraduate medical programme is offered in a predominantly Arab-Islamic environment and is the first co-educational programme at the national university, delivered on an otherwise gender-segregated campus. Our medical students enter directly from secondary school and generally are 18–20 years of age when starting PHCPs. The majority of students are female, some of whom have been raised in strict gender-segregated environments.

Clinical skills programmes, reported as involving weekly bedside teaching, longitudinal mentoring, early contact with patients and problem-based or case-based learning, improve students’ preparedness for the first clerkship [8]. The authors assert, however, that although students initially value working with simulated patients in the clinical skills lab, they eventually crave more clinical integration affording more opportunities to apply their medical knowledge to real patients (ibid). Further, several studies from the UK and Europe contend that students express a preference for a gradual transition into clinical environments [8–11].

The majority of undergraduate medical programmes in the Gulf Cooperative Council (GCC) comprise preclinical and clerkship phases. ECE is usually limited to clinical lectures and learning of clinical skills in laboratory and simulation settings, [12] delaying clinical exposure in healthcare settings until the clerkship phase. Unusual in the Middle East, we have introduced early clinical placements in primary care from the onset of the MD programme. We anticipated that early PHCPs would be beneficial to students by easing the transition to clinical clerkships in future years and providing early exposure to a co-educational, culturally-diverse, clinical environment.

Bourdieu [13] contends that tertiary education cannot be studied in isolation, but rather, must be examined within a broader social sphere. Educational models developed elsewhere do not always take the cultural mores and values of the new environment into consideration and may incorporate practices that do not map onto the new social terrain. Fahy’s [14] study of Moroccan university students, contends that educational models developed in the West do not necessarily transition into the Moroccan socio-political milieu because the skills cultivated at university do not conform, or even clash with societal expectations. Kane’s [15] ethnography documents the inherent difficulties of transplanting a Western medical programme to Qatar and challenges presumptions of universality of medical knowledge built into medical education. Qatari society adheres to a relatively conservative view of proper comportment of the sexes. It is worth noting that the physical layout of domestic houses include male and female-only spaces, the primary health care centres and public buildings have gender-segregated waiting areas and that Qatar University has separate male and female campuses. Several studies in Western contexts report feelings of shame amongst medical students in their clinical training [16–18]. A study of Jordanian doctors and nurses reveals that they activate “Islamic self-surveillance” during their work [19]. Arab students from allied health professions revealed shyness and shame in their first year of clinical practice due to discussing personal, intimate and body issues, revealing a need for educational frameworks to help overcome these feelings [20]. A study of the impact of shame on clinical nursing education contends shame has a detrimental effect on learning, consequently addressing shame in medical education is essential [21]. To circumvent these issues, the clinical faculty worked in collaboration with a team of local family physicians to develop this PHCP programme. This combined expertise helped develop learning outcomes likely to be achieved and informed the delivery of the programme in a culturally-sensitive manner.

Inherent benefits of ECE are clearly documented particularly when it involves clinical placements. A study from Iran contends that medical students recognise that early ECE strengthened their learning in the pre-clinical phase and was regarded as an opportunity to familiarise themselves with doctoring skills and patient care [22]. Another study explored two cohorts of Indonesian students’ experiences during the first clerkship rotation; one trained exclusively in a skills laboratory and the other having undertaken a short clinical skills programme in PHCPs in the pre-clerkship phase [23]. Their study showed that ECE in the PHCPs built students’ confidence, enhanced interpersonal communication skills and clinical reasoning, preparing them better for the first clerkship. Focus groups with 4th year medical students who had weekly contact with patients in hospital outpatient clinics during the previous year, conclude that ECE ameliorates the shock of transitioning into medical practice [11].

Concerns are nonetheless expressed by several authors on the effects of first clinical exposure on medical students [8, 22, 24]. ECE may be daunting and stressful to young students who may lack the maturity to cope with these clinical encounters. Medical students’ stress and anxiety associated with entering clinical clerkships has been previously documented [8, 24]. Similarly, a recent study of year 4 medical students entering their first clerkship in Sharjah, U.A.E., reports 59% (*N* = 37) of the cohort experienced high stress and 75% (*N* = 46) admitted finding the first few weeks difficult [25]. Given the established link between stress and the commencement of clerkships, it stands to reason that clinical exposure at an earlier age may compound these anxieties. Clinical placements immerse students in an unfamiliar organisational culture imbued with new language, hierarchies, procedures, and concepts of healthcare systems centered on the patient and their family, provided by a team of health care professionals with different roles and responsibilities. Consequently, this may elicit a broad range of feelings amongst the novice medical students, including but not limited to excitement and feelings of burden.

This study examines the interplay between novice medical students’ experiences of ECE and their identity development. The primary objective of the study was to explore medical students’ perceptions of early clinical exposure in PHCPs supported by ER sessions, and to identify challenges encountered. Specifically, the research assessed the students’ perceptions about the aims, their clinical learning and barriers to learning during the PHCPs and their learning during the ER sessions. A secondary aim of this study was to explore differences in these perceptions between second and third year medical students and any differences reported between males and females.

## Methods

### Study design, setting and participants

A new ECE programme was introduced in a new medical school in Qatar University in 2016. Students commence the MD program at year 2 after completing one academic year of foundation studies. In the PHCP programme, initial visits to the PHCPs provide students with an overview of the services available to patients in the primary care setting. From second semester students commence performing history taking and clinical examination of patients under the guidance of clinical faculty preceptors. Each clinical faculty preceptor is responsible for a pair of students attending the PHCPs. Occasionally, if they were not available to supervise students, other doctors in the Health Centre provided supervision. Clinical education is taught alongside an organ systems-based, problem-based learning programme which focuses on acquisition of the basic medical sciences from year 2. Students learn clinical skills on simulated patients in the clinical skills lab for 2 h weekly and attend monthly PHCP visits in pairs for 3 h to hone these skills on real patients throughout the 2.5 year pre-clerkship phase. In addition to clinical skills acquisition, the CMED programme of ECE is designed to reinforce non-technical skills. PHCP learning outcomes were organised with a focus on exploring how patient care works, how the healthcare system functions and how care delivery is achieved. In addition, students observed how different healthcare providers interact with each other and with the patient, and how clinical knowledge and guidelines are applied in practice. These learning outcomes are made available to students and clinical faculty via sets of questions to encourage a sense of inquiry, intended to enhance observation of situations and encounters in alignment with desired learning outcomes.

To support students attending PHCPs, students in small groups participate in experiential review (ER) sessions, facilitated by a behavioural scientist and a family physician at regular intervals. Students deconstruct their clinical encounters and reflect on their PHCP experiences. These sessions provide important scaffolding by providing a safe forum to discuss any professional, personal or ethical issues students are contemplating. ER sessions offer space to explore different meanings, reflect on personal experiences from the PHCPs, and help discuss various interpretations that foster the development of desired professional identities. They are also opportunities to clarify clinical decision-making and raise concerns arising from observing healthcare delivery to a culturally-diverse population.

This evaluation reports experiences of students in the first two and a half years of the MD program (i.e. second and third years). Students were placed in six different health centres and attended monthly PHCP visits in pairs for 3 h visits. A cross-sectional survey was conducted as an evaluation of the ECE programme components at the end of each academic year from 2017 to 2019, to elicit student feedback on their experiences and which enabled improvements to be made. Selected on the basis of their participation in PHCPs and ER, all second and third year medical students were invited to complete an on-line questionnaire using the “Survey Monkey” platform. Students were invited to participate on a voluntary basis at their own convenience. All second and third year students were eligible and there were no exclusion criteria. As the survey was conducted via an online-electronic route, the students were encouraged to participate using an email reminder. To further encourage participation and reduce desirability bias, students were assured that their participation was anonymous, and their personal data were not required during the data collection.

### Questionnaire

The questionnaire was based on the aims of the programme developed by university and primary care faculty and their achievement. The survey had separate sections on demographics and student perceptions about: the aims of the PHCPs and the learning that ensued from the opportunities afforded; their confidence in performing clinical tasks and barriers encountered to learning clinical care. As the questionnaire was developed with the intention of strengthening components of the programme, we asked students to reply honestly with any concerns that if found general, would be acted on. The questionnaire took approximately ten minutes to complete**.**

The questionnaire is shown below:

**Table:** 

**Primary Health Care Clinical Placement Questionnaire** 1. The purpose of clinical placements in the Primary Healthcare Center is to: A. See as many patients as possible B. Learn about how care is provided to patients C. See a multidisciplinary team of healthcare professionals in action D. Learn about the different providers of healthcare within the PHC Centre such as doctors, nurses, pharmacist, lab workers, etc. E. Practise history taking and clinical examination on real patients under supervision F. Practise interpreting clinical tests G. Observe doctors as role models H. See patients with medical problems we study in our PBL cases I. Discuss with family physicians the management of common clinical problems J. Observe ethical and professional dilemmas 2. How well do the clinical placements allow you to: A. See as many patients as possible B. Learn about how care is provided to patients C. See a multidisciplinary team of healthcare professionals in action D. Learn about the different providers of healthcare within the PHC Centre such as doctors, nurses, pharmacist, lab workers, etc. E. Practise history taking and clinical examination on real patients under supervision F. Practise interpreting clinical tests G. Observe doctors as role models H. See patients with medical problems we study in our PBL cases I. Discuss with family physicians the management of common clinical problems J. Observe ethical and professional dilemmas 3. The purpose of the experiential review session is: A. To review and share cases seen in PHC Centre B. Discuss any ethical and professional dilemmas consequent on the PHC visits C. To review history taking and examination skills D. To develop our communication skills further E. To discuss any difficulties we are having in the clinical environment F. To learn clinical medicine 4. How well does the experiential learning session allow you to: A. To review and share cases seen in PHC Centre B. Discuss any ethical and professional dilemmas consequent on the PHC visits C. To review history taking and examination skills D. To develop our communication skills further E. To discuss any difficulties we are having in the clinical environment F. To learn clinical medicine Responses to Questions 1–4 are: Strongly Agree/ Agree/ Not Sure/ Disagree/ Strongly Disagree 5. Please describe what fits your situation best: A. I can confidently take histories and examine patients; I improved my skills in the PHC clinical placements B. I can confidently take histories and examine patients; I didn’t improve my skills in the PHC clinical placements C. I cannot confidently take histories and examine patients; however, I have improved my skills in the PHC clinical placements D. I cannot confidently take histories and examine patients; and I have not improved my skills in the PHC clinical placements E. I cannot confidently take histories and examine patients; and I do not often attend PHC clinical placements 6. What do you think are barriers to you learning about clinical care in the PHCPs? Please tick all answers that apply to you: A. The faculty are too busy to teach B. I learn when the faculty are available but do not learn as much from other doctors I am placed with C. The faculty do not seem interested in my learning needs D. I think I am often too shy to ask for the experience I need in the PHC centre E. I find learning with students of the opposite sex difficult and this detracts from my learning F. I find talking to patients still difficult and a bit embarrassing G. I feel I am too young to be exposed to the clinical environment at present H. I feel that I lack an understanding of clinical medicine and consequently I don’t find the clinical environment as useful as I might do later in my training I. I don’t find it useful learning from other healthcare professionals (I learn only from doctors) J. I am not assertive enough to gain the opportunities I need in the clinical environment K. The learning outcomes for the clinical placements in each unit are not clear L. There are not enough patients in the PHC centre for me to see	

Patients or the public were not involved in the design, or conduct, or reporting, or dissemination plans of our research.

### Primary and secondary outcome measures

The primary outcomes were the students’ perceptions of the aims of the PHCP programme and of their own learning during the PHCPs shown in the questionnaire. Secondary outcome measures were perceived barriers to clinical learning. We asked whether students agreed with the aims of the PHCPs and the ER sessions and determined how effectively they were implemented. Further sections explored students’ confidence in developing their clinical skills and barriers to clinical learning experienced.

### Sample size

The total number of eligible students enrolled in the MD programme during the years 2017–2019 were 191 in second year and 116 in third year. Since this evaluation was done using an online survey where response rate tends to be poor, a decision was made to include every response in order to improve representativeness of the students’ perceptions.

### Data analysis

Data analysis was carried out using R Statistical software and graphs in Microsoft Excel. All data were categorical and were summarised using frequencies and percentages. Comparisons between second year and third year students were all done using chi-squared tests or exact tests, when there were small expected frequencies. A *p*-value < 0.05 was deemed significant.

### Ethics

This evaluation was reviewed by Qatar University Institutional Review Board and the QU IRB number is QU-IRB 005-NR/20. Each student opted voluntarily to complete the anonymous questionnaire and data reported cannot be attributed back to individual students.

## Results

A total of 151 (50.3%) out of the eligible 300 students completed the questionnaire, 107 (70.9%) were in second year and 44 (29.1%) in third year (Table 1). There were three cohorts of students studied so it is likely that some students completed the survey in subsequent years, however, as this was an anonymous survey their responses cannot be directly compared. Seventy-three percent of the respondents were females. Most students identified as Qatari (27.2%) or originating from an Arab country (61.2%). By third year, most students were at least 20 years old.

**Table 1:** Demographic characteristics of students completing the surveys

		Overall *N* = 151	Year 2 *N* = 107	Year 3 *N* = 44
Gender, n (%), (*N* = 148)	Male	41 (27.3)	30 (28.3)	11 (25.0)
	Female	109 (72.7)	76 (71.7)	33 (75.0)
Year of survey, n (%), (N = 151)	2017	39 (25.8)	39 (36.4)	–
	2018	49 (32.5)	29 (27.1)	20 (45.5)
	2019	63 (41.7)	39 (36.4)	24 (54.5)
Ages, n (%), (*N* = 146)	< 20 years	51 (34.9)	47 (44.8)	4 (9.8)
	≥20 years	95 (65.1)	58 (55.2)	37 (90.2)
Ethnicity, n (%), (*N* = 147)	Qatari	28 (27.2)	23 (27.4)	5 (26.3)
	Other Arab countries	63 (61.2)	49 (58.3)	14 (73.7)
	Rest of world	12 (11.2)	12 (14.3)	–

### Students perceptions about the purpose of the clinical placements and what they learnt

**Fig. 1:**
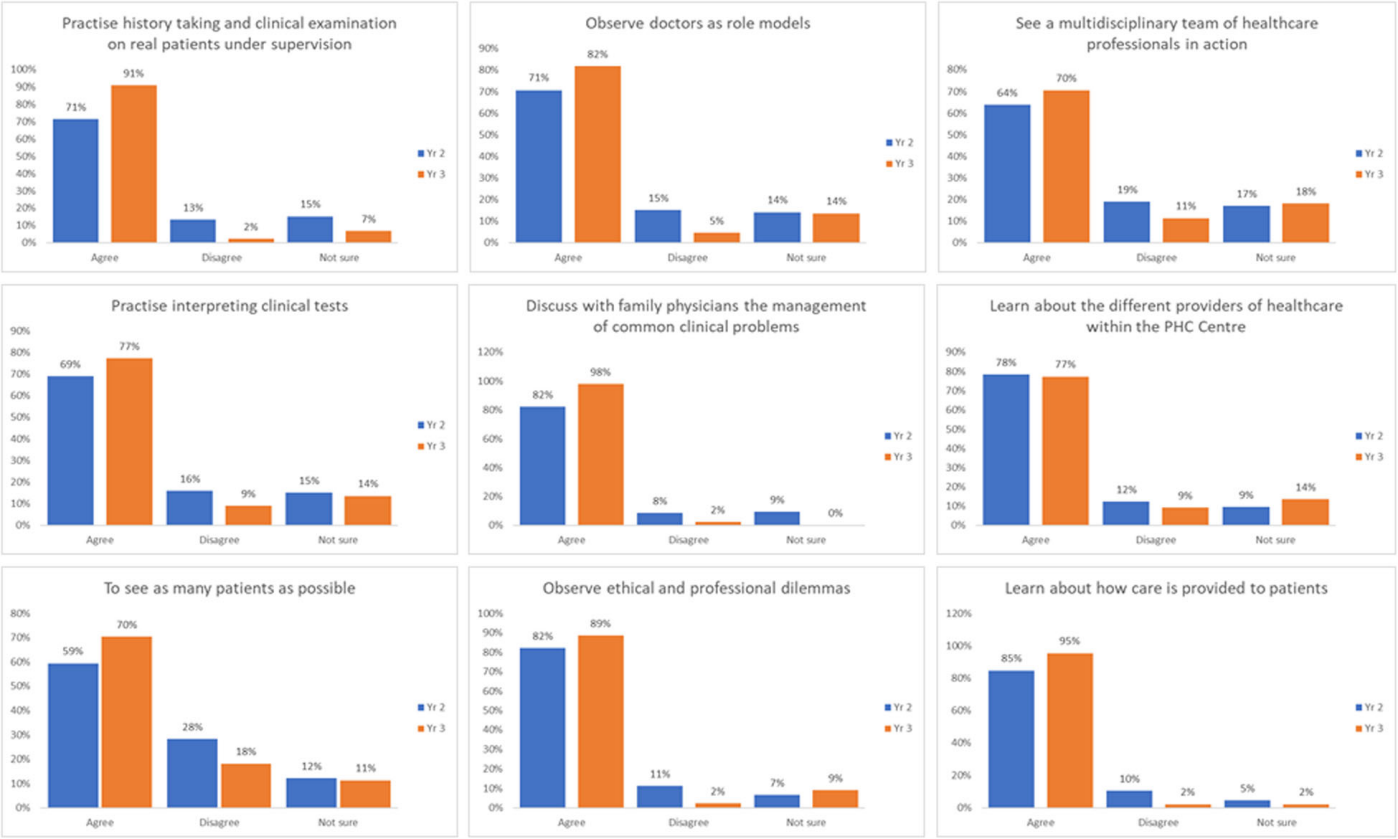
Student perceptions about the purpose of the clinical placements

Figure 1 summarises the students’ perceptions on whether they agree with the aims of the PHCPs. Both second year and third year students were generally in agreement with the intended aims of the PHCPs, with most either strongly agreeing or agreeing.

**Fig. 2:**
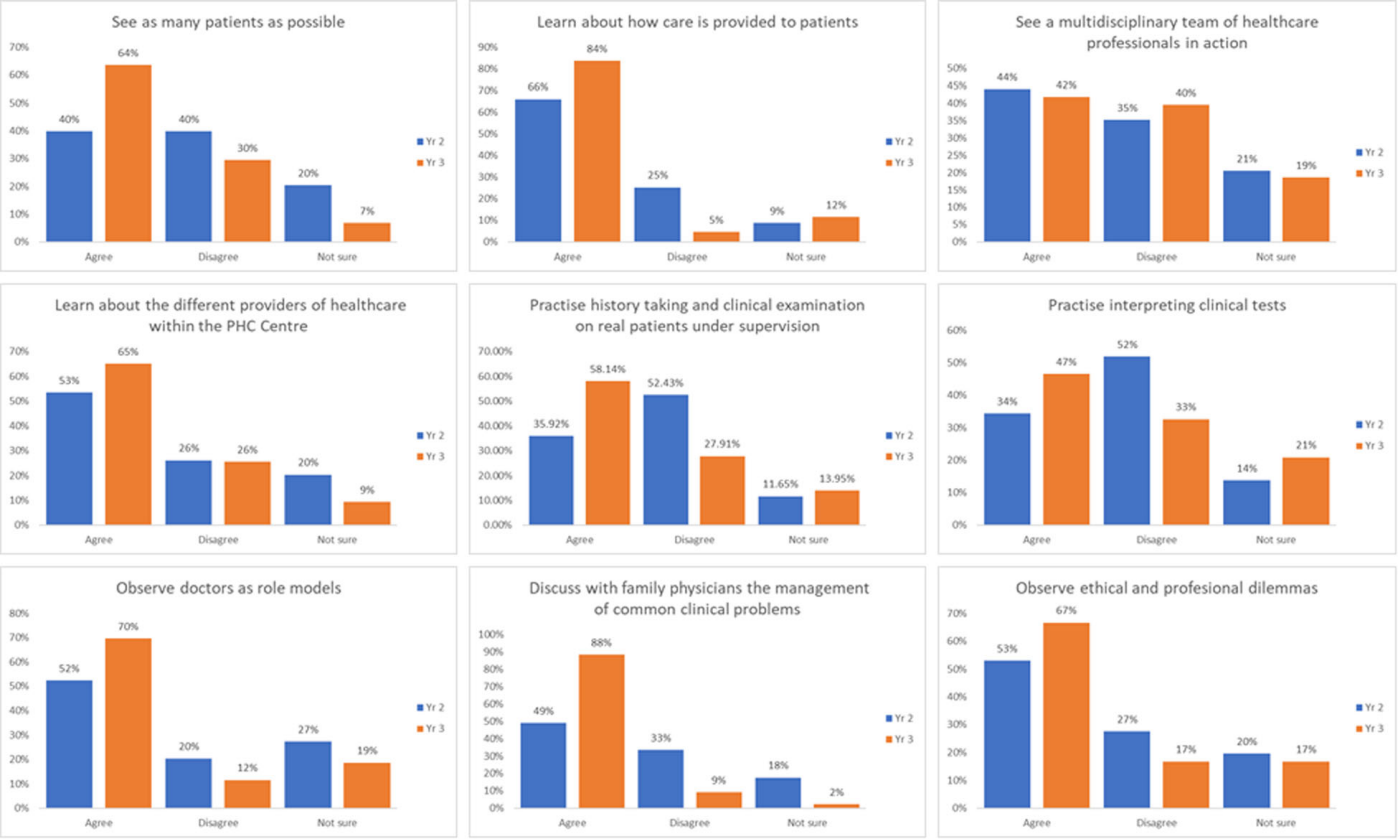
Students' perceptions about what the PHCPs allowed them to do

Figure 2 summarises students’ perceptions about what they learned during the clinical placements. The supplementary table combines student perceptions about the purpose of clinical placements with what they learned during the placements.

Most of the students either strongly agreed or agreed with the majority of the statements with the exception of two statements. Students were ambivalent that the clinical placement allowed them to see a multidisciplinary team of healthcare professionals in action and to practise interpreting clinical tests. Compared to students in second year, students in the third year were significantly more likely to either strongly agree or agree that PHCPs afforded opportunities to: see as many patients as possible (*p* = 0.042), learn about how care is provided to patients (*p* = 0.043), see patients with medical problems they studied in their case units (*p* < 0.001) and learn about how care is provided to patients in primary care (p = 0.043) (Supplementary Table & Fig. 2).

### Student perceptions of their confidence in performing clinical tasks

**Fig. 3:**
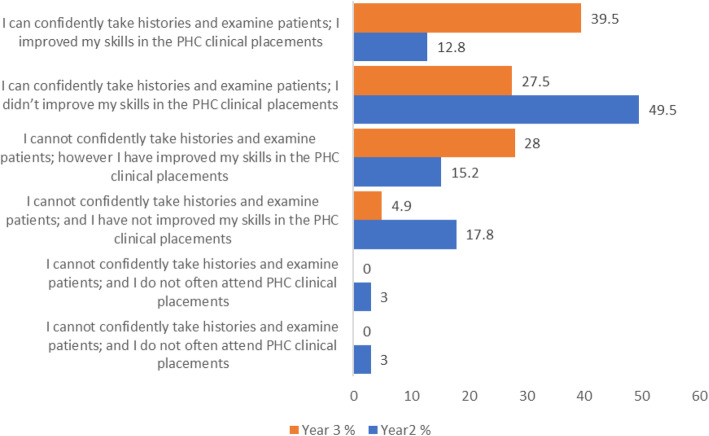
Students perceptions about their confidence at performing tasks during the PHCPs

Students were asked about their confidence at performing tasks during the PHCPs. (Fig. 3).

Most of the students (year 2 = 62.5%, year 3 = 67%) report being confident in their ability to take histories and examine patients. Third year students were more likely to attribute their skills improvement to experience acquired in the clinical placements.

### Students’ perceptions of barriers to learning clinical care in PHCPs

Table 2 summarises students’ perceived barriers to learning about clinical care in the PHCPs.

**Table 2:** Students’ perceptions of barriers to learning clinical care in PHCPs

Year of Survey	Overall *N* = 151	Year 2 *N* = 107	Year 3 *N* = 44	*p*-value	Female *N* = 107	Male *N* = 41	*p*-value
The faculty are too busy to teach	63 (41.7)	45 (42.1)	18 (40.9)	0.897	43 (40.2)	20 (48.8)	0.344
I learn when the faculty are available but do not learn as much from other doctors I am placed with	41 (21.2)	30 (28.0)	11 (25.0)	0.703	31 (29.0)	10 (24.4)	0.577
The faculty do not seem interested in my learning needs	37 (24.5)	29 (27.1)	8 (18.2)	0.247	27 (25.2)	10 (24.4)	0.916
I think I am often too shy to ask for the experience I need in the PHCP	40 (26.5)	26 (24.3)	14 (31.8)	0.341	30 (28.0)	10 (24.4)	0.655
I find learning with students of the opposite sex difficult and this detracts from my learning	9 (5.7)	6 (5.6)	3 (6.8)	0.775	5 (4.7)	4 (9.8)	0.247
I find talking to patients still difficult and a bit embarrassing	39 (25.8)	27 (25.2)	12 (27.3)	0.795	23 (21.5)	16 (39.0)	**0.030**
I feel I am too young to be exposed to the clinical environment at present	18 (11.9)	16 (15.0)	2 (4.6)	0.073	12 (11.2)	7 (17.1)	0.340
I feel that I lack an understanding of clinical medicine and consequently I don’t find the clinical environment as useful as I might do later in my training	44 (29.1)	33 (30.8)	11 (25.0)	0.473	30 (28.0)	14 (34.2)	0.467
I don’t find it useful learning from other healthcare professionals (I learn only from doctors)	15 (9.9)	12 (11.2)	3 (6.8)	0.412	10 (9.4)	5 (12.2)	0.607
I am not assertive enough to gain the opportunities I need in the clinical environment	23 (15.2)	11 (10.3)	12 (27.3)	**0.008**	14 (13.1)	9 (22.0)	0.183
The learning outcomes for the clinical placements in each unit are not clear	73 (48.3)	58 (54.2)	15 (34.1)	**0.025**	48 (44.9)	25 (61.0)	0.079
There are not enough patients in the PHCP for me to see	60 (39.7)	48 (44.9)	12 (27.3)	**0.045**	47 (43.9)	13 (31.7)	0.175

The most common barriers to clinical learning were the clinical faculty being too busy to teach (41.7% students), the learning outcomes being unclear (48.3%) and a perceived lack of patients at the PHCPs for the students to see (39.7%). Compared to those in Year 2, students in the third year were significantly more likely to perceive that they were not assertive enough to gain opportunities to learn during PHCP (27.3% vs 10.3%, *p* = 0.008). However, compared to students in the third year, students in the second year were significantly more likely to perceive that the learning outcomes for the PHCP were not clear (54.2% vs 34.1%, *p* = 0.025) and to perceive that there were not enough patients in the PHCP for them to see (44.9% vs 27.3%, *p* = 0.045). With respect to gender, male students were significantly more likely to report that they found talking to patients still difficult and embarrassing, compared to female students (39.0% vs 21.5%, *p* = 0.030).

### Students’ perceptions of the purpose and their learning during the ER sessions

Table 3 summarises students’ understandings of the purpose of the Experiential Review sessions.

**Table 3:** Students’ perceptions of the purpose and their learning during the ER

		The purpose of the ER session is:				The experiential learning session allowed me to:			
Question	Response	Total (%)	Year 2 (%) *N* = 104	Year 3 (%) *N* = 44	*p*-value	Total (%)	Year 2 (%) *N* = 104	Year 3 (%) *N* = 44	*p*-value
To discuss any difficulties, we are having in the clinical environment	Strongly Agree and Agree	60.3	56.5	70.4	0.078	49.4	41.4	68.1	**0.002**
	Not sure	18.9	18.3	20.5		20.3	26.0	6.8	
	Strongly Disagree and Disagree	20.3	25.0	9.1		30.4	42.7	25.0	
Discuss any ethical and professional dilemmas consequent on the PHCPs	Strongly Agree and Agree	80.4	75.9	90.9	0.075	70.2	66.4	79.6	0.146
	Not sure	11.5	14.4	4.5		12.2	15.4	4.5	
	Strongly Disagree and Disagree	8.1	9.6	4.5		17.6	18.3	15.9	
To review history taking and examination skills	Strongly Agree and Agree	83.1	79.8	90.9	0.206	73.6	68.2	86.3	**0.043**
	Not sure	9.5	10.6	6.8		10.8	14.4	2.3	
	Strongly Disagree and Disagree	7.5	9.6	2.3		15.6	17.3	11.3	
To develop our communication skills further	Strongly Agree and Agree	27.1	27.9	25.0	0.909	17.6	18.2	15.9	0.805
	Not sure	20.9	20.2	22.7		19.6	18.3	22.7	
	Strongly Disagree and Disagree	52.0	51.9	52.3		62.9	63.2	61.4	

Overall, the majority of students in both years either strongly agreed or agreed with the stated objectives of the ER sessions apart from the statement that ER sessions allowed them to develop their communications skills further.

## Discussion

Student feedback supports ECE as beneficial to our students in Qatar: Students saw a range of patients and learned about the delivery of primary care in PHCPs. They discussed managing common medical conditions with family physicians and witnessed ethical and professional dilemmas. Students attribute improvements in patient history taking and examination skills to exposure to the clinical environment, particularly in third year. ER provided a forum to discuss ethical and professional dilemmas and difficulties students were having in the clinical environment. Our findings, though new to the region, are consistent with reports of the benefits of ECE in Europe, North America and South East Asia [5, 6, 23, 26]. ECE in primary healthcare environments has been commended by students and their clinical faculty: Benefits extend beyond experiential learning of clinical skills to encompass increased student awareness of patient autonomy and improvement in their communication skills [23]. A recent study compared experiences of two groups of first year medical students applying basic sciences to a clinical case: One experienced an ECE setting and the other undertook a traditional lecture format [26]. Students in the ECE cohort developed ethics, attitude, and professionalism, as observed in our study.

PHCPs were added to our clinical education programme after the original approval of the MD programme assessment framework and consequently students’ performance in these placements is not assessed summatively. The medical programme is based on self-directed learning providing scope for students to engage in activities of benefit. As a result, students were initially required to attend a minimum of 75% of all PHCP sessions as per university policy. From 2018 these sessions were made mandatory as a consequence of the evaluation which showed their benefit.

As this was a nascent programme, annual student evaluations enabled faculty to make improvements. Student feedback initially indicated learning outcomes were unclear and suggested clinical faculty were too busy to teach. These issues have been described as prevalent in clinical teaching elsewhere [27]. We have since developed handbooks to ensure clarity of learning outcomes. As the programme has expanded we increased clinical faculty from 15 to 70, utilised more primary health centres and appointed a family physician as a liaison between the university and the Primary Health Care Corporation responsible for the PHCPs. This expansion of clinical faculty necessitated a faculty-development programme to familiarise doctors with the learning outcomes. The family physician acting as liaison visited each health centre to observe and support clinical teaching and sent out explicit instructions by email to the clinical faculty at the beginning of each week of PHCPs.

In the Middle East, ECE involving clinical placements is not yet an established educational paradigm, consequently there was little information to inform the design and implementation of our clinical programme. Aware that sociocultural factors may hinder students’ ability to interact with patients, we recognised the importance of providing a safe forum to discuss their anxieties and concerns surrounding patient encounters. During clinical training students are exposed to patients from different cultural backgrounds and acquire new forms of knowledge that may be perceived as deviating from customary expectations. A study of the other medical college in Qatar documents how the faculty presumed wrongly that its students understood the sociocultural norms of the West, necessary to provide appropriate medical care to this significant patient group attending the PHCPs [28]. Our ER discussions aimed to help contextualise and normalise issues raised by the students after their visits to the PHCPs.

More than a quarter of students report talking to patients to be difficult and embarrassing. Secondly, some students contend that learning with students of the opposite sex is difficult and detracts from their learning (5.7%). There may be a number of possible factors contributing to these findings. First, our students are novice learners, as such they may be overwhelmed as they assume their changing professional identities in the clinical environment. Second, cultural differences may influence their interactions. Discussions during ER sessions revealed that although students recognise that clinical necessity requires them to elicit information of a highly personal nature and to interact with all sexes, some students expressed concerns that these medical encounters contravene societal norms and values. During ER sessions it emerged that some students engage in a degree of self-surveillance and some experience magnified feelings of shame when they perform clinical skills involving actions such as: touching bodies, maintaining eye contact and asking patients of the opposite sex routine questions about smoking, alcohol consumption and sexual history. Similar concerns were articulated by medical students in Qatar a decade earlier [28]. Our students also raised an issue not addressed in the PHCP literature which may emanate from the sociocultural context of the Arabian Gulf: 10% do not recognise the value of learning from other healthcare professionals, preferring instead to only learn from doctors. On one hand, this may indicate that some students do not recognise the value of interprofessional learning. Over 50% of students acknowledged, however, that PHCPs allowed them to learn about the different providers of healthcare. Alternatively, students may have lacked meaningful, deliberate experiences with other healthcare colleagues in the PHCPs. An additional factor may stem from enduring professional and ethnic hierarchies at play as physicians in the region are generally accorded high status, most of whom are Arab or trained in the West. Clearly, medicine training is embedded within different socio-economic and cultural contexts, each bringing different issues to the fore.

Although there were clearly articulated concerns from students about their limited clinical knowledge, their shyness and lack of assertiveness to seize learning opportunities; and some embarrassment and difficulty in talking to patients, these issues affected only the minority. Prince and colleagues [10] studied student preparedness for clinical practice in 71 fourth year medical students at Maastricht University. Almost half were nervous at the beginning of the clerkship; 8.4% students felt afraid to start a conversation with a patient and only 22.9% felt their knowledge sufficient. Nineteen percent of students had had no previous real patient contact [10]. These align with our findings, although real clinical exposure is earlier, our students are younger, and are raised in Arab-Islamic cultures.

One question that arises, is whether or not it is too early to send medical students to PHCPs in the early years, particularly where students have entered medical college directly from school? When asked directly, fewer than 1 in 8 of the students indicated they felt too young to be exposed to the clinical environment (15% in year 2 and 4.6% in year 3). In a medical school in Utrecht which also admits students directly from secondary education, students complete ‘early’ internal medicine and surgery clerkships in third year [29]. The authors report early clerkships are advantageous and that neither young age, nor limited clinical knowledge impede successful completion of the early clerkships. Although the authors do not report on anxiety or on barriers to clinical learning, they conclude that students, like the majority of ours, value ECE. A study from the UK compared anxiety at the transition into clerkships in two groups of students who started medical school: those directly entering from school (mean age 19 years) and graduates (mean age 25.5 years) [30]. The graduates were found to be less anxious and more prepared than the undergraduates upon entry to clerkships. These findings were attributed not only to maturity, but also to the mode of delivery of the graduate entry medical programme involving early introduction of clinical skills teaching in parallel with a fully integrated clinically relevant curriculum with continued assessment (similar to our undergraduate programme structure), whereas the undergraduates experienced a traditional medical school programme. Themes identified as concerning to both groups on entering clerkships were uncertainty about their roles, lack of clinical skills experience, perceived difficulty integrating factual knowledge into clinically useful information and a concern about the depth of knowledge required. Similar concerns also find traction in our Middle Eastern setting, suggesting that these are universal. Nonetheless, the question we pose here is, are these novice students’ perceptions manifesting underlying cultural sensitivities or are they acculturating to their new environment? We recommend further exploration of students’ feelings and emotions of such experiences to better understand them and uncover links to sociocultural dimensions.

## Conclusions

Preliminary data support the introduction of early clinical placements for medical students in the Middle East as part of a clinical education programme. Most students benefitted from ECE: they learned about the delivery of clinical care to patients and gained familiarity with the clinical environment. Most students reported confidence in history and examination skills, and by the end of third year many attribute an improvement in history and examination skills to the PHCPs. Introducing early PHCPs for novice medical students, in conjunction with supportive university-based teaching and learning through ER debriefing sessions alongside a robust clinical skills’ programme fosters learning and clinical confidence in an Arab setting.

## Supplementary Information

**Additional file 1 Supplementary Table.** Students’ perceptions about the purpose of clinical placements and what they learned during the placements.

## Data Availability

The datasets used and/or analysed during the current study are available from the corresponding author on reasonable request.
