# Supplementary material for: A program evaluation reporting student perceptions of early clinical exposure to primary care at a new medical college in Qatar
Source: BMC Med Educ. 2021 Mar 17;21:162. doi: 10.1186/s12909-021-02597-9 (PMC7968227; doi:10.1186/s12909-021-02597-9)
Supplement: Supplementary file 1 — Additional file 1 Supplementary Table. Students’ perceptions about the purpose of clinical placements and what they learned during the placements. [file 12909_2021_2597_MOESM1_ESM.docx]

**A Program Evaluation Reporting Student Perceptions of Early Clinical Exposure to Primary Care at a New Medical College in Qatar**

Tanya Kane^1^, Tawanda Chivese^1^, Ayad Al-Moslih^2^, Noora A M Almutawa^3^, Suhad Daher-Nashif^1^, Nehdia Hashemi^2^, Alison Carr*^2^

1. Department of Population Medicine, College of Medicine, QU Health, Qatar University, Doha, Qatar
2. Department of Clinical Academic Sciences, College of Medicine, QU Health, Qatar University, Doha, Qatar
3. Primary Health Care Corporation, Doha, Qatar

*Corresponding author: Professor Alison Carr,

Associate Dean for Clinical Education

Address: College of Medicine, Qatar University
P.O. Box 2713
Doha, Qatar

Email: a.carr@qu.edu.qa

Tel: +974 44037809

### **Supplementary Table:** Students’ perceptions about the purpose of clinical placements and what they learned during the placements

|  |  | The purpose of clinical placements in the PHCP is to: | | | | The clinical placements in the PHCP allowed me to: | | | |
| --- | --- | --- | --- | --- | --- | --- | --- | --- | --- |
| Question | Response | Total, N = 151 | Year 2, N=107 | Year 3, N = 44 | p-value | Total, N = 151 | Year 2, N = 107 | Year 3, N = 44 | p-value |
|  |  | % | % | % |  | % | % | % |  |
| See as many patients as possible | Strongly Agree | 21.2 | 20.6 | 22.7 |  | 11.6 | 9.7 | 15.9 |  |
|  | Agree | 41.7 | 39.3 | 47.7 | 0.661 | 35.4 | 30.1 | 47.7 |  |
|  | Not sure | 11.9 | 12.1 | 11.4 |  | 16.3 | 20.4 | 6.8 | 0.042 |
|  | Disagree | 18.5 | 21.5 | 11.4 |  | 25.2 | 25.2 | 25.0 |  |
|  | Strongly Disagree | 6.6 | 6.5 | 6.8 |  | 11.6 | 14.6 | 4.5 |  |
| Learn about how care is provided to patients | Strongly Agree | 40.0 | 34.9 | 52.3 |  | 17.1 | 14.6 | 23.3 |  |
|  | Agree | 48.0 | 49.1 | 45.5 |  | 54.1 | 51.5 | 60.5 |  |
|  | Not sure | 4.0 | 4.7 | 2.3 | 0.096 | 9.6 | 8.7 | 11.6 | 0.043 |
|  | Disagree | 5.3 | 7.5 | 0.0 |  | 11.6 | 16.5 | 0.0 |  |
|  | Strongly Disagree | 2.7 | 3.8 | 0.0 |  | 7.5 | 8.7 | 4.7 |  |
| See a multidisciplinary team of healthcare professionals in action | Strongly Agree | 24.7 | 24.5 | 25.0 |  | 9.7 | 8.8 | 11.6 |  |
|  | Agree | 41.3 | 39.6 | 45.5 |  | 33.8 | 35.3 | 30.2 |  |
|  | Not sure | 17.3 | 17.0 | 18.2 | 0.710 | 20.0 | 20.6 | 18.6 | 0.921 |
|  | Disagree | 10.0 | 12.3 | 4.5 |  | 24.1 | 22.5 | 27.9 |  |
|  | Strongly Disagree | 6.7 | 6.6 | 6.8 |  | 12.4 | 12.7 | 11.6 |  |
| Learn about the different providers of healthcare within the PHC Centre such as doctors, nurses, pharmacist, lab workers, etc | Strongly Agree | 25.8 | 24.3 | 29.5 |  | 11.0 | 9.7 | 14.0 |  |
|  | Agree | 52.3 | 54.2 | 47.7 |  | 45.9 | 43.7 | 51.2 |  |
|  | Not sure | 10.6 | 9.3 | 13.6 | 0.759 | 17.1 | 20.4 | 9.3 | 0.472 |
|  | Disagree | 6.6 | 6.5 | 6.8 |  | 16.4 | 17.5 | 14.0 |  |
|  | Strongly Disagree | 4.6 | 5.6 | 2.3 |  | 9.6 | 8.7 | 11.6 |  |
| Practise history taking and clinical examination on real patients under supervision | Strongly Agree | 36.7 | 32.1 | 47.7 |  | 11.0 | 5.8 | 23.3 |  |
|  | Agree | 40.7 | 39.6 | 43.2 |  | 31.5 | 30.1 | 34.9 |  |
|  | Not sure | 12.7 | 15.1 | 6.8 | 0.094 | 12.3 | 11.7 | 14.0 | 0.011 |
|  | Disagree | 4.7 | 5.7 | 2.3 |  | 18.5 | 20.4 | 14.0 |  |
|  | Strongly Disagree | 5.3 | 7.5 | 0.0 |  | 26.7 | 32.0 | 14.0 |  |
| Practise interpreting clinical tests | Strongly Agree | 29.1 | 28.0 | 31.8 |  | 11.7 | 10.8 | 14.0 |  |
|  | Agree | 42.4 | 41.1 | 45.5 |  | 26.2 | 23.5 | 32.6 |  |
|  | Not sure | 14.6 | 15.0 | 13.6 | 0.548 | 15.9 | 13.7 | 20.9 | 0.287 |
|  | Disagree | 8.6 | 8.4 | 9.1 |  | 22.8 | 24.5 | 18.6 |  |
|  | Strongly Disagree | 5.3 | 7.5 | 0.0 |  | 23.4 | 27.5 | 14.0 |  |
| Observe doctors as role models | Strongly Agree | 27.2 | 21.5 | 40.9 |  | 15.8 | 13.6 | 20.9 |  |
|  | Agree | 47.0 | 49.5 | 40.9 |  | 41.8 | 38.8 | 48.8 |  |
|  | Not sure | 13.9 | 14.0 | 13.6 | 0.094 | 24.7 | 27.2 | 18.6 | 0.408 |
|  | Disagree | 8.6 | 11.2 | 2.3 |  | 10.3 | 11.7 | 7.0 |  |
|  | Strongly Disagree | 3.3 | 3.7 | 2.3 |  | 7.5 | 8.7 | 4.7 |  |
| Discuss with family physicians the management of common clinical problems | Strongly Agree | 30.7 | 29.0 | 40.0 |  | 16.6 | 13.7 | 23.3 |  |
|  | Agree | 48.0 | 49.5 | 40.0 |  | 37.9 | 34.3 | 46.5 |  |
|  | Not sure | 10.2 | 9.3 | 15.0 | 0.628 | 17.9 | 19.6 | 14.0 | 0.151 |
|  | Disagree | 7.1 | 7.5 | 5.0 |  | 20.0 | 22.5 | 14.0 |  |
|  | Strongly Disagree | 3.9 | 4.7 | 0.0 |  | 7.6 | 9.8 | 2.3 |  |
| Observe ethical and professional dilemmas | Strongly Agree | 15.4 | 15.4 | NA |  | 73.9 | 73.9 | NA |  |
|  | Agree | 59.0 | 59.0 | NA |  | 26.1 | 26.1 | NA |  |
|  | Not sure | 7.7 | 7.7 | NA |  |  |  |  |  |
|  | Disagree | 10.3 | 10.3 | NA |  |  |  |  |  |
|  | Strongly Disagree | 7.7 | 7.7 | NA |  |  |  |  |  |
